# ProphNet: A generic prioritization method through propagation of information

**DOI:** 10.1186/1471-2105-15-S1-S5

**Published:** 2014-01-10

**Authors:** Víctor Martínez, Carlos Cano, Armando Blanco

**Affiliations:** 1 Department of Computer Science and AI, University of Granada, Granada, 18071, Spain

## Abstract

**Background:**

Prioritization methods have become an useful tool for mining large amounts of data to suggest promising hypotheses in early research stages. Particularly, network-based prioritization tools use a network representation for the interactions between different biological entities to identify novel indirect relationships. However, current network-based prioritization tools are strongly tailored to specific domains of interest (e.g. gene-disease prioritization) and they do not allow to consider networks with more than two types of entities (e.g. genes and diseases). Therefore, the direct application of these methods to accomplish new prioritization tasks is limited.

**Results:**

This work presents ProphNet, a generic network-based prioritization tool that allows to integrate an arbitrary number of interrelated biological entities to accomplish any prioritization task. We tested the performance of ProphNet in comparison with leading network-based prioritization methods, namely rcNet and DomainRBF, for gene-disease and domain-disease prioritization, respectively. The results obtained by ProphNet show a significant improvement in terms of sensitivity and specificity for both tasks. We also applied ProphNet to disease-gene prioritization on Alzheimer, Diabetes Mellitus Type 2 and Breast Cancer to validate the results and identify putative candidate genes involved in these diseases.

**Conclusions:**

ProphNet works on top of any heterogeneous network by integrating information of different types of biological entities to rank entities of a specific type according to their degree of relationship with a query set of entities of another type. Our method works by propagating information across data networks and measuring the correlation between the propagated values for a query and a target sets of entities. ProphNet is available at: http://genome2.ugr.es/prophnet. A Matlab implementation of the algorithm is also available at the website.

## Background

The advancements in high-throughput technologies such as DNA sequencing, linkage analysis, association studies and expression arrays have fostered the research towards an effective personalized medicine. To this end, the integration of pieces of evidence of different nature derived from diverse data sources is required, together with algorithms able to mine these data and identify novel biological facts of relevance. Networks have been shown to be an useful representation for combining heterogeneous biological data. Currently, there is a huge availability of large molecular networks such as protein-protein interaction (PPI) networks, which model interactions between proteins. Many methods have been proposed in the literature to represent and mine knowledge from biological networks [1]. For example, [2] proposes to apply text-mining in OMIM to generate a similarity network for human diseases and [3] builds a gene network based on the results of microarray experiments. These approaches have led to the emergence of new methods that exploit and integrate different data sources into networks in a variety of ways [4]. Inferring new knowledge from existent networks is usually based on "guilt-by-association" [5]. This extensively validated principle states that biological entities which are associated or interacting in a network are more likely to share a common function. This principle allows to infer new relationships from already known interactions.

In this context with massive amounts of highly interconnected data is where prioritization methods are required. Prioritization tools are based on computational approaches that use information retrieved from diverse sources in order to obtain ranked lists of candidate biological elements (genes, proteins, diseases, etc.) related with a certain target element. Gene-disease prioritization, in which genes are ranked according to their relevance to a disease of interest (or vice versa), is the most popular prioritization task, and many methods have been proposed in the recent literature to accomplish this task [6]. Most of these methods focus on the analysis of phenotype and PPI networks for gene-disease prioritization. These methods weight the arcs connecting two proteins or phenotypes according to a measure of the similarity between them. CIPHER [7] computes correlation coefficients based on linear regressions of phenotype and PPI profiles. PRINCE [8] computes the relevance of a gene by using network propagation methods. RWRH [9] scores genes and diseases using a random walk approach on PPI and phenotypes networks. rcNet [10] proposes a methodology for prioritization of candidate genes based on propagating node values and measuring the degree of correlation between two sets of nodes, one in the PPI/gene network and one in the phenotype network. Network-based gene-disease prioritization methods have been proven to provide better results than previous approaches [11-15].

Apart from gene-disease prioritization, other methods have been proposed to perform a prioritization of other biological entities. DomainRBF [16] performs a prioritization of protein domains for diseases using Bayesian linear regression. This method assumes a key role for protein domains in diseases as shown by previous studies [17]. Domains are basic structural and functional units of proteins, which in turn are composed of multiple structural domains, each one closely linked to a specific function. Although DomainRBF exploits the functional role of protein domains in phenotypes, it does not explore the simultaneous integration of PPI, domain and phenotype networks for gene or disease prioritization. Despite the good performance obtained by the mentioned prioritization methods, they have clear limitations. First, existing network-based prioritization methods do not allow to consider more than two types of networks for performing the prioritization (e.g. gene and disease networks in rcNet and domain and disease networks in domainRBF). Only non-network-based methods have succeeded in integrating more than two different types of entities for prioritization. For example, Endeavour [13] performs an independent prioritization of different entities using multiple heterogeneous generic data sources which are integrated on a single global ranking using order statistics. However, previously mentioned network-based methods have been shown to outperform this method using a lower amount of data sources [7].

Second, existing prioritization methods are strongly tailored to a specific domain of interest (for example gene-disease prioritization for rcNet and protein domain-disease prioritization for domainRBF, respectively). In our opinion, these two drawbacks dramatically limit the applicability of these methods to other prioritization tasks and do not allow to improve the results by integrating information about other types of related entities.

In this work we present ProphNet, a generic prioritization method that outperforms previous methods by integrating and propagating information in an arbitrary number of heterogeneous data networks. Our method is generic since it allows to prioritize biological entities of any type with respect to biological entities of another type. Therefore, the user can customize the goal of the prioritization task (disease-gene, domain-disease, drug-disease, etc.). Furthermore, the user is not restricted to the use of only two entities, and can integrate as many biological networks as desired.

**Figure 1:**
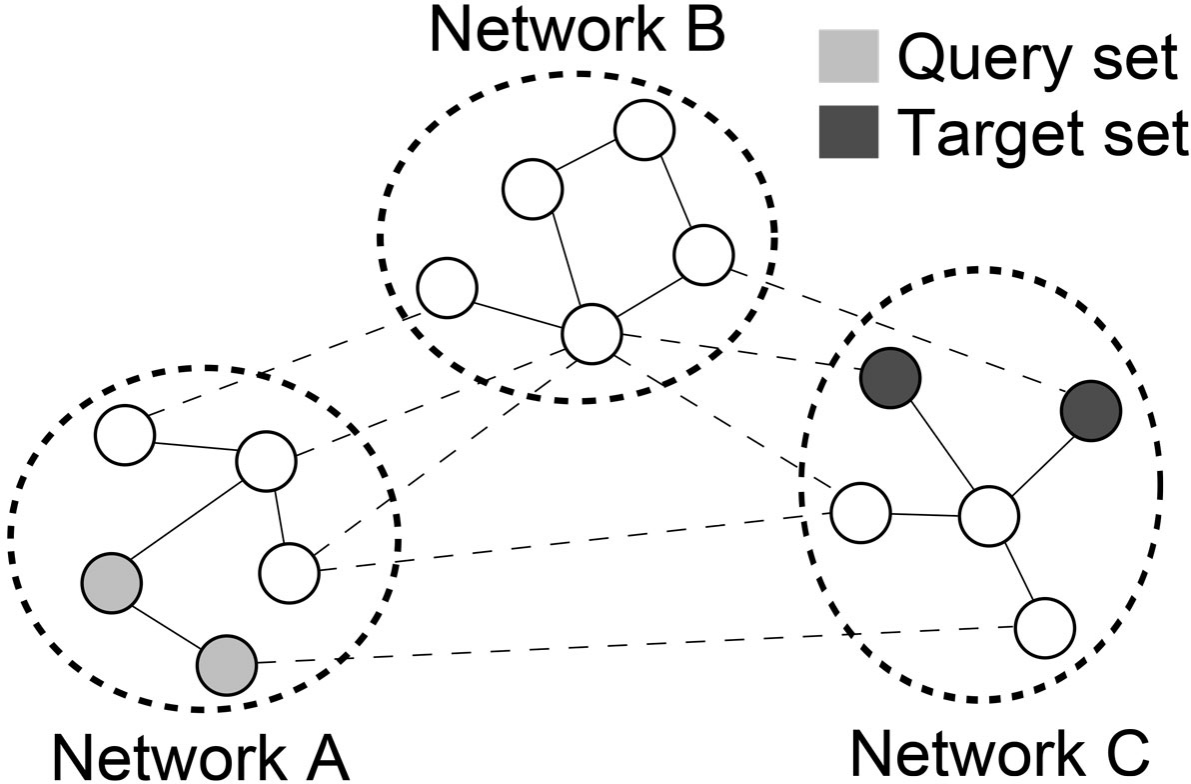
**Problem Overview**. Our problem is to determine how related the query set and the target set are based on known relations between elements.

To compare the results obtained by ProphNet with those obtained by state-of-the-art methods, such as rcNet and domainRBF, we applied ProphNet to the prioritization of genes-diseases and domains-diseases, respectively, on a network built as the integration of protein domain, PPI and phenotype networks. ProphNet measures the influence of a query set of biological entities of a certain type (e.g. genes or diseases) in a target set of entities of another type (e.g. diseases or genes, respectively). To this end, the algorithm uses a graph representation as shown in Figure 1. In this representation, each node corresponds to a biological entity of a domain of interest (gene/protein, disease, protein domain, etc.), and the arcs between two nodes are labelled with a weight representing the strength of the relationship between the connected entities. These weights are derived from different databases and other biological sources and their interpretation varies depending on the type of the connected entities and the final goal of the study (e.g. physical/structural similarity, regulatory dependence, similar functional roles, etc.). In our algorithm, the arc weights for each network are compiled in an adjacency matrix. The nodes of the graph are also labelled with a value (in [0, 1]), representing the degree of association to the query or the target set. There are two kinds of networks: a) networks representing interactions or similarities between entities of the same type, and b) networks representing interactions or similarities between entities of different type. Type b) networks are used to interconnect type a) networks.

The method we propose allows to propagate node values through paths along different data networks (representing different biological entities) in order to derive new information from the existing knowledge. This value propagation is performed in two directions. First, values are propagated within and between networks, through all the possible paths connecting the query set network to the target set network (not reaching the target set network). Second, values are also propagated within the target set network, starting from the target nodes. Both propagation processes follow the principle that the higher the weight of the arc between two entities is, the more similar the value of these two nodes should be. Therefore, these two label propagation processes derive a final graph in which the value assigned to a node represents its degree of relation with the query or target set, respectively. Finally, we measure the degree of relationship between the query and target sets by computing the correlation between the values assigned to the nodes in the target network and those assigned to their neighbour nodes in other networks, as proposed in previous works with good results for different prioritization tasks [7,10]. This process is explained in detail in the following section.

This article is organized as follows. The method and the data sources are described in detail in section *Methods*. To validate the proposed methodology we integrate protein domain, PPI and phenotype networks and compare the results to those obtained by rcNet for gene prioritization and DomainRBF for domain prioritization. These results are presented in the *Results *section and show a significant improvement in terms of sensitivity and specificity. ProphNet is also applied to several case studies (namely Alzheimer, Diabetes Mellitus Type 2 and Breast Cancer) to identify putative candidate genes involved in these diseases. The results of these tests can be found in the section *Case Studies*. Finally, some conclusions and future work are presented.

## Methods

Let *D *be a set of graphs (also referred to as networks) defined as *D_i _*= (*V_i_, E_i_*) for *i *= 1, ..., *n*, where *V_i _*is a set of vertices which represent biological entities from a specific domain satisfying *V_i _∩ V_j _*= ∅, ∀*i, j *such that *i *≠ *j*. Each node *v_ik _*(with *k *= 1, ..., *|V_i_|*) in *D_i _*is labelled with a value Ψ(*v_ik_*), initially set to zero, that indicates the degree of relationship to the query or target set, depending on the network *v_ik _*belongs to. *E_i _*is a set of weighted undirected arcs representing relationships, similarities or interactions between elements of *V_i_*. The values of the nodes change while the weights of the arcs remain constant during the entire process. Let *R *be a set of graphs defined as *R_ij _*= (*V_i _*∪ *V_j_, C_ij_*), where *C_ij _*is a set of weighted undirected arcs representing relationships, similarities or interactions between elements of *V_i _*and *V_j_*, with *i, j *∈ 1, ..., *n *and *i *≠ *j*. Therefore, *R_ij _*describes the relationships between the biological entities from two different networks: *D_i _*and *D_j_*.

We define the heterogeneous global graph *G *as *G *= (*D, R*). Let the graph *D_q _*∈ *D *be the query network and let *D_t _*∈ *D *be the target network. Given the global graph *G*, our goal is to find the degree of association between a set of nodes *Q *⊆ *V_q _*called the query set and a set of nodes *T *⊆ *V_t _*called the target set.

The initial values for the nodes in the query set are set to 1 (Ψ(*v_qi_*) = 1 for all nodes *v_qi _*∈ *Q*), while the rest of the nodes are set to 0 (Ψ(*v_qj_*) = 0 for nodes *v_qj _*∈ *V_q _- Q*). The target network is initialized in the same way, but considering the nodes in *V_t _*and *T*. The rest of nodes in *G *are initially set to 0.

As we explain below in more detail, our method performs a propagation within networks pumping information between nodes. This process is based on the Flow Propagation algorithm [18,19], which uses the normalized Laplacian matrix to propagate labels between nodes in a network. The normalization takes into account the degree of each node to limit the bias toward annotations from high-degree nodes. This normalization is also critical for convergence. The Flow Propagation algorithm is similar to a Random Walk with Restart, basically differing in the normalization process that guides the propagation [19].

Let *N *be the non-normalized adjacency matrix of a network in *G*. Since *G *= (*D, R*) and graphs in *R *are bipartite (i.e. the adjacency matrices of graphs in *R *are not squared), let assume *N *has *r *rows and *c *columns. A normalization for *N *can be computed as:

$$norm\left(N\right)=D_{G}^{1}ND_{G}^{2},$$

where $D_{G}^{1}$ and $D_{G}^{2}$ are diagonal matrices where each component is defined as:

$$D_{G_{jj}}^{1}=\frac{1}{\sqrt{\left(\sum_{k=1}^{c}N_{jk}\right)}}$$

for *j *= 1, .., *r*, and

$$D_{G_{kk}}^{2}=\frac{1}{\sqrt{\left(\sum_{j=1}^{r}N_{jk}\right)}}$$

for *k *= 1, .., *c*.

We define *M *= {*M_i _| M_i _= norm*(*D_i_*) where *i *= 1, .., |*D*|} as the set of normalized squared adjacency matrices of graphs in *D*. Similarly, we define *S *= {*S_i _| S_i _= norm*(*R_i_*) where *i *= 1, .., *|R|*} as the set of normalized adjacency matrices of bipartite graphs in *R*.

**Figure 2:**
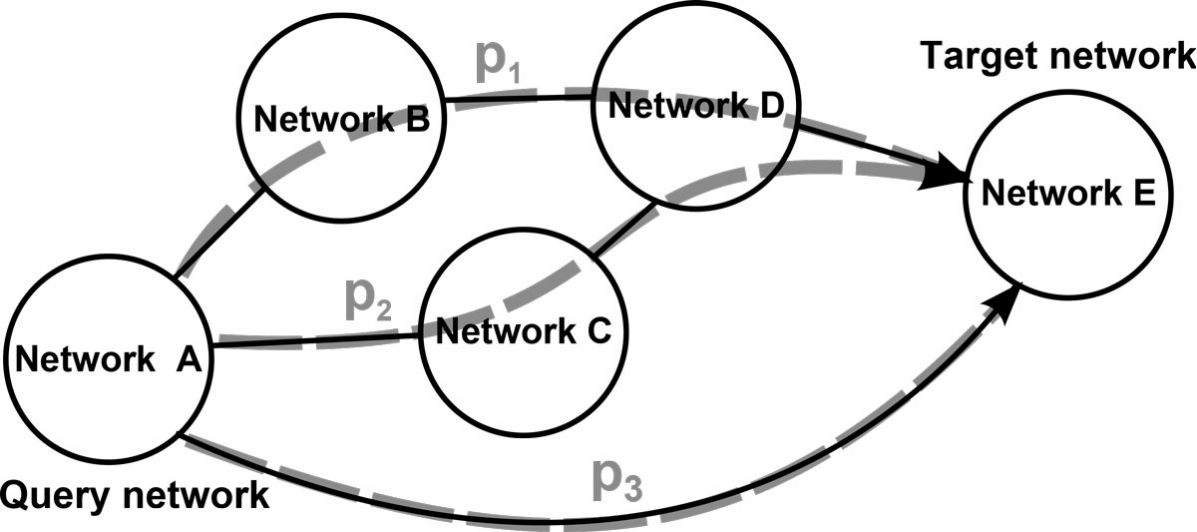
**Example of path computation**. Example of computed paths. Three paths have been obtained connecting the query network to the target network.

Let *p_i _*= {*p*_*i*1_, ..., *p_ij_*, ..., *p_il_*} be a path composed of networks from *D *connecting *D_q _*and *D_t_*, satisfying *p_ij _*∈ *D, p*_*i*1 _= *D_q_, p_il _= D_t _*and *p_ij _*≠ *p_ik_*, ∀*j *≠ *k*. To compute the degree of association between the query and target sets, we first propagate values from the query set within the query network, and from the target set within the target network, as described in Section *Value propagation inside networks*. Next, we identify all the possible paths *P *= {*p*_1_, ..., *p_|P|_*} connecting the query network to the target network. Figure 2 shows an example of a global graph *G *composed of five different networks or domains, with three different paths connecting the query network to the target network. Since the number of networks is usually reduced, the computation of all the paths connecting *D_q _*and *D_t _*can be accomplished by a brute force algorithm. For each computed path *p_i_*, we propagate information from *p_ij _*to the following network *p*_*i*(*j*+1) _in the path, as described in Section *Value propagation between networks*. Then we propagate information within the network *p*_*i*(*j*+1)_, where *j *= 1, 2, ..., *l *- 2. The propagation continues until it has been performed within the network *p*_*i*(*l-*1) _in the path.

Finally, after performing this propagation through each path in P, we correlate the values of the nodes in *D_t _*against the values of the nodes in *p*_*i*(*l-*1) _directly connected to those in *D_t_*, for all the paths *p_i _*∈ *P*. This step is described in Section *Value correlation between networks*. The obtained correlation value determines the degree of relationship between the query set and the target set.

Although measuring the degree of relationship between the query and target sets by correlating node values seems less intuitive than continuing the propagation of node values from the neighbours networks to the target nodes, the former approach has been shown to perform better than the latter (see Additional file 1). Therefore, it was selected as the measure of similarity in our method. This approach was proposed in previous network-based prioritization methods with good results for different prioritization tasks [7,10]. In order to accomplish prioritization tasks, in which only the query set *Q *∈ *V_q _*and the target network *V_t _*are provided by the user, we embed this pipeline into an iterative process to score each node in the target network according to its relationship with *Q*. This process is described in Section *Prioritization process*.

### Value propagation inside networks

Several propagation methods have been proposed to compute the similarity or distance between nodes within a graph [4]. Methods based on local neighbourhood or shortest paths fail in capturing global interactions, in contrast to global methods that take into account the entire network topology [20]. ProphNet implements a flow propagation approach [18,19] that uses a network's global information to perform a propagation of the values assigned to the nodes within this network. To carry out this propagation process within a network *D_k_*, we first define the prior information set *Z *as those vertices *v_kj _*with Ψ(*v_kj_*) ≠ 0. Therefore, the prior information set matches *Q *when propagating values within the query network, and the prior information set matches *T *when propagating values within the target network. The value Ψ(*v_kj_*) of each node *v_kj _*in *Z *(*j *∈ [1, *|V_k_|*]) is normalized as:

$$\Psi \left(v_{kj}\right)=\frac{\Psi \left(v_{kj}\right)}{\sum^{v_{kx}\in V_{k}}\Psi \left(v_{kx}\right)}$$

Let *x*_0 _be a vector compiling the values initially assigned to each node in *D_k_*, and $\hat{x}$ a vector with the values assigned to each node in *D_k _*after performing the propagation within *D_k_*. To calculate $\hat{x}$ we need to solve the following optimization problem:

$$\underset{\hat{x}}{\min}\underset{i,j}{\sum }M_{k_{i,j}}{\left({\hat{x}}_{i}-{\hat{x}}_{j}\right)}^{2}+\frac{1-\alpha }{\alpha }\underset{i}{\sum }{\left({\hat{x}}_{i}-x_{0_{i}}\right)}^{2}$$

where *M_k _*is the network's normalized adjacency matrix. The closed-form solution of this equation is:

$$\hat{x}=\left(1-\alpha \right){\left(I-\alpha M_{k}\right)}^{-1}x_{0}.$$

This linear system can be solved exactly. However, there exists an iterative algorithm for solving this system which is faster for large networks [21]:

$$x_{i+1}=\alpha M_{k}x_{i}+\left(1-\alpha \right)x_{0}$$

with *i *starting from 0. This algorithm implements an iterative process where each node propagates its node value to its neighbours, based on the weights of the arc connecting them. The parameter *α *∈ [0, 1] determines the importance of the prior information set.

In order to further speed up this iterative process, we define the following stopping criterion: *|x_i _*- *x*_*i*+1_| ≤ *κ*. This allows to stop the iterative process when it has almost converged, without the need of full convergence. Experimental tests (results not shown) prove that the best performance is obtained for *κ *≤ 10^*-*3^.

For convenience, we refer to ${\hat{x}}_{kj}$ as the vector obtained after convergence, where each component represents the value assigned to each node in the network *D_k _*after performing the propagation within *D_k_*, as part of a propagation process through the path *p_j_*. Since the propagation values within the query and target networks are not affected by the propagation processes through the paths in *P*, we define ${\hat{x}}_{q}$ as the vector obtained after propagating nodes values within the query network, and ${\hat{x}}_{t}$ as the vector obtained after propagating nodes values within the target network.

### Value propagation between networks

Given a network *D_i _*whose vertices are already assigned a value according to ${\hat{x}}_{il}$, we further propagate these values to the next network *D_j _*in the current path *p_l_*, with *D_j _*≠ *D_t_*. Since *D_i _*and *D_j _*are connected by *R_ij_*, the information is propagated from the nodes of *D_i _*to the nodes of *D_j _*across the edges of *R_ij _*by assigning each node *v_jk _*from *D_j _*a value computed as the mean of the nodes from *D_i _*the node *v_jk _*is connected with. This expression is formalized as:

$$\Psi \left(v_{jk}\right)=\frac{\sum^{v_{ix}\in neig_{i}\left(v_{jk}\right)}\Psi \left(v_{ix}\right)}{\left|neig_{i}\left(v_{jk}\right)\right|}$$

where *neig_i_*(*v_jk_*) is the set of nodes from *D_i _*that are directly connected to *v_jk _*according to *R_ij_*. A thresholding step is applied to this propagation process, since detailed experimentation suggested that nodes with very low values add noise to the process and reduce the performance (see Additional file 2). To this end, a parameter *γ *∈ (0, 1] is included in the process so that the ⌈*|V_j_|*(1 *- γ*)⌉ lowest node values from *D_j _*after the propagation are updated to Ψ(*v_jk_*) = 0. The rest of the node values are not changed.

### Value correlation between networks

After the propagation process through one path finishes, the nodes in the networks which are adjacent to the target network present values that determine their degree of relationship to the query set. Also, the nodes in the target network are assigned a value that determines the degree of relationship with the target set. We can indirectly measure the relationship between the query set and the target set by measuring the similarity between the values of the nodes in the target network and those that are directly connected to them in adjacent networks. This can be calculated by simultaneously correlating these node values as derived by the propagation processes through all the different paths. For each path *p_i _*with length *l *a vector ${\bar{x}}_{i}$ is computed as:

$${\bar{x}}_{i}=S_{a}{\hat{x}}_{\left(l-1\right)i}$$

where *S_a _*is the normalized adjacency matrix of *R*_(*l-*1)(*l*) _and ${\hat{x}}_{\left(l-1\right)i}$ is the vector obtained after propagating values inside the network *D*_*l-*1_.

Since the values of the target network after the propagation process are represented by ${\hat{x}}_{t}$, we define the vector $\bar{t}$ as:

$$\bar{t}=concat\overset{|P|\text{ times}}{\overset{︷}{\left({\hat{x}}_{t},\ldots ,{\hat{x}}_{t}\right)}}$$

and the vector $\bar{x}$ as:

$$\bar{x}=concat\left({\bar{x}}_{i}\right)\;\forall i\in \left[1,|P|\right]$$

where *concat *means concatenation. Both $\bar{x}$ and $\bar{t}$ are the same size.

Finally, the correlation value which derives a measure for the relationship between the query and target sets is computed as:

$$s=corr\left(\bar{x},\bar{t}\right)$$

where *corr *is Pearson's Correlation.

### Prioritization process

In order to obtain a prioritized list of targets for a query set of nodes, we have to follow an iterative approach. For each target network node *v_te_*, we set it as the target set *T *and compute the correlation value *s *as described in the previous section (we called this correlation *s_e _*since it is computed for *T *= {*v_te_*}). Once this correlation value has been computed for each target network node, these nodes are sorted in decreasing order according to their *s_e _*value. Target nodes with high values of *s_e _*are supposed to be strongly related to the query set. The entire algorithm is described in the pseudocode *Algorithm 1*

**Algorithm 1 **ProphNet

**prioritize(***G*: global graph, *Q*: query set, *D_q_*: query network, *D_t_*: target network)

   Propagate values within *D_q_*

   *P*: Compute the list of paths from *D_q _*to *D_t _*in *G*

   **for each **path *p_i _*= {*p*_*i*1_, ..., *p_ij_*, ..., *p_il_*} **in ***P ***do**

      **for each **network *p_ij _***in **the path *p_i _*from *p*_*i*1 _to *p*_*i*(*l-*1) _**do**

         Propagate values from *p_ij _*to *p*_*i*(*j*+1)_

         Propagate values within *p*_*i*(*j*+1)_

      **end for**

      Store the values of *D*_*i*(*l-*1) _after propagation through path *p_i _*as ${\hat{x}}_{i\left(l-1\right)}$

   **end for**

   **for each **entity *e *∈ *V_t _*in the target network *D_t _***do**

      Set target set *T *= {*e*}

      Propagate values within *D_t_*

      Compute correlation coefficient *s_e _*using the stored ${\hat{x}}_{i\left(l-1\right)}$ for each path *p_i_*.

   **end for**

   *L*: Sort all entities *e *∈ *V_t _*by their *s_e _*values in descending order

   **return ***L*

### Prioritization example

**Figure 3:**
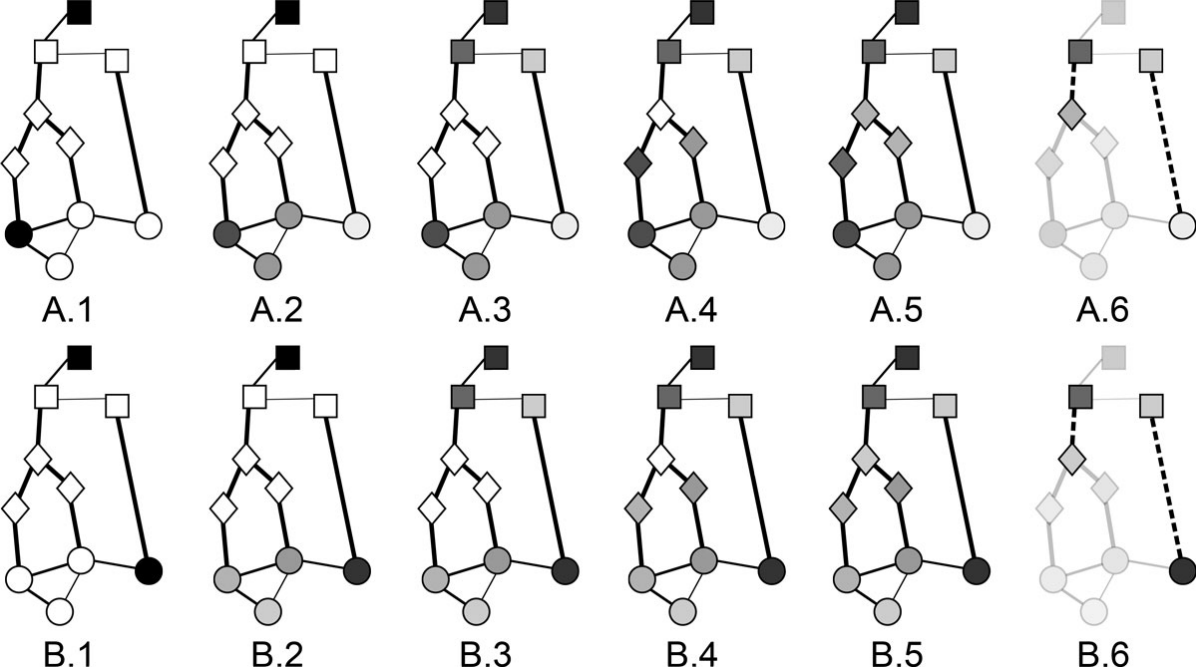
**Examples of step-by-step ProphNet runs**. Step-by-step runs of ProphNet in two global graphs for the same target set but different query sets. Figure (a) shows an example in which propagated values from the query and target sets show a high correlation and therefore they seem to be related. In figure (b) propagated values from the query and target sets show low correlation, thus suggesting a weak relationship.

To facilitate the understanding of the algorithm, the Figure 3 shows a step-by-step representation of the ProphNet propagation processes. This figure shows two examples of a prioritization task involving three networks or domains, with the elements of each network represented by circles, squares and diamonds, respectively. For simplicity and clarity, node values are represented using a grey color scale (from white representing value 0 to black representing value 1) and the weight of an arc is represented by its line width. In the two examples, the prioritization task involves the same target set but different query sets. The query and target sets contain only one element in both cases, which is initially (step 1) set to 1 (black). Node values are propagated from the query nodes within the query network (step 2), and from the target nodes within the target network (step 3). There are two paths connecting the query network and the target network in these examples (circles-squares and circles-diamond-squares, respectively). Values from the query network are then iteratively propagated to adjacent non-target networks. Since the query network is directly connected to the target network in one of the paths, this step (step 4) is only applied to the path which includes an intermediate network (diamonds). Then, the propagation is performed within this intermediate network (step 5). This propagation continues until all the networks in all the paths connecting the query and target sets have been reached. Finally, we measure the strength of the connection between the query and the target sets as the correlation between the values assigned to the nodes in the target network and the values assigned to their neighbours from other networks (step 6).

Figure 3a shows an example in which the values propagated from the query and target sets are highly correlated, suggesting a strong relationship between them. On the other hand, Figure 3b shows an example with low correlation values, which suggests that query and target sets are not related.

### Algorithm complexity

The time complexity of the algorithm shown in the pseudocode *Algorithm 1 *can be determined by aggregating the time complexity of each task it is composed of. Let *n *be the number of nodes in a network and *m *the number of networks in the global graph *G*. The task of propagating values within a network is *O*(*n*^3^). The propagation of values between networks is *O*(*n*^2^). The computation of the correlation coefficient for one path is *O*(*n*^3^). The number of paths is bounded by *m*! and their length by *m*. Therefore, the computational complexity of ProphNet is bounded by *O*(*m*! *× m × n*^3^). Despite this high complexity, typical execution times are a few seconds since the value of *m *is usually small in real applications. A summary of ProphNet execution times and memory usage for the experiments shown in this paper can be found in Additional file 3.

## Results

As two specific case studies, we have applied ProphNet to prioritize candidate genes and protein domains associated to diseases. ProphNet has been compared with rcNet for gene-disease prioritization and with DomainRBF for domain-disease prioritization, since these methods were recently proposed and reported better results than previous methods [10,16]. ProphNet was run on a global graph composed of diseases, genes and protein-domains interconnected networks, while rcNet and DomainRBF were run on a genes-diseases and domains-diseases networks, respectively, as proposed by their authors. It is important to note that the ProphNet base case execution using only the gene and disease networks would obtain the same results than rcNet. The data sources used are described in detail in Section *Data sources*.

We tested the performance of the different methods on several leave-one-out (LOO) cross-validation experiments and for predicting new associations recently added to OMIM. To measure the performance of the different prioritization methods, we used Receiver Operating Characteristic (ROC) curves. ROC curves plot the true positive rate vs. the false positive rate at various threshold settings. The area under the ROC curve (AUC) was also computed. Finally, the average ranking position of the true target in the prioritized lists obtained by each method was also computed and normalized by dividing by the total number of elements in the list (5080 diseases in this case). We also computed p-values for comparing the average ranking values using two-tailed t-tests and the Bonferroni correction.

For the results reported for ProphNet, *α *was set to 0.9, the error threshold in the flow propagation was set to *κ *= 10^*-*5 ^and *γ *= 0.00375. For rcNet, we set the parameters to the values providing better results according to the authors: *α *= 0.9, *β *= 0.9 and *κ *= 10^*-*5 ^[10] and used the enumeration-correlation based version.

### Data sources

The disease phenotype network has been extracted from OMIM [22] using text mining techniques as described in [2]. Also, to perform a fair comparison of the results to those reported by rcNet, we used a version of OMIM from May, 2007 [10]. The obtained disease network contains 5080 OMIM disease phenotypes. The arcs are weighted with a value in the range [0, 1]. This weight measures the similarity between two phenotypes as the inverse of the distance between the feature vectors obtained by counting the occurrences of each term from the anatomy and disease sections of the Medical Subject Headings Vocabulary (MeSH) in the description text for the corresponding entries in OMIM. The obtained disease network contains a total of 19,729 weighted interactions.

The gene network has been obtained from the Human Protein Reference Database (HPRD [23]). This protein-protein interaction network contains 32,331 unique interactions between 8,919 proteins. The network connecting genes and phenotypes has been extracted directly from OMIM (phenotype-gene relationship fields) obtaining 1,393 relationships.

The domain network has been derived from DOMINE [24] and InterDom [25] containing 48,778 unique relations between 5,490 domains. Relations between domains and genes were extracted from pFam [26]. Relations between domains and phenotypes have been extracted from Pfam and annotations of nsSNPs in the UniProt database [27].

The three networks (genes, protein domains and diseases) have simultaneously been used in the experiments performed with ProphNet. RcNet was executed using only the gene and disease networks, since this method does not allow to integrate more than two networks. DomainRBF was run on the domain and disease networks due to the same limitations.

### Gene-disease validation

To check whether the prioritization methods rcNet and ProphNet were able to retrieve a known relationship between a gene and a disease, we performed a leave-one-out cross-validation using gene-phenotype relations from OMIM. For each gene-phenotype relation reported in OMIM, we run the two algorithms on a network in which the explicit arc connecting the gene and phenotype of interest was removed. The gene of interest was set as the query set and the methods were asked to rank all the phenotypes associated to this query set.

**Figure 4:**
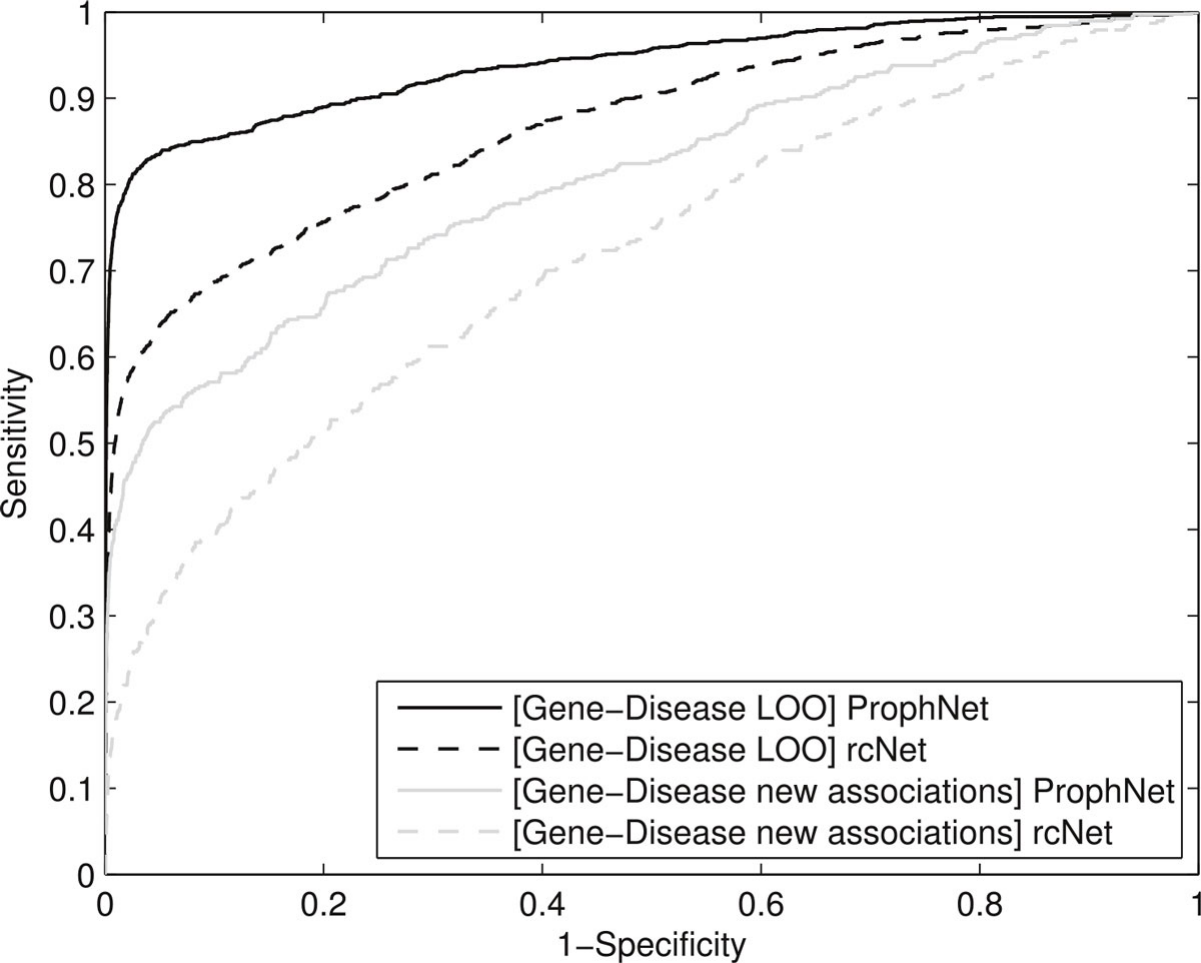
**ROC curves ProphNet vs. rcNet**. ROC curves for gene-disease prioritizations with ProphNet and rcNet.

The obtained ROC curves are shown in Figure 4. AUC values and avg. rank values for the target disease are displayed in Table 1. We can see that ProphNet outperforms rcNet in this test, ranking the target phenotype in a significantly higher position (corrected p-value *<*0.05), with lower standard deviation and obtaining better AUC values. The high difference in terms of AUC value (over 10%) also suggests that the achieved improvement is not due to ProphNet prioritizing a little better those targets poorly prioritized by rcNet, but these targets being prioritized at the top by our method while they are poorly prioritized by rcNet. It is also important to note that, although a high percentage of the cases were prioritized in the top of the ranking, we found some results that were really worse ranked by both methods, significantly increasing the mean ranking and setting it far from the top 1 position. This also applies to experiments described in the following two sections.

**Table 1:** Tests results

Test	Method	AUC	Normalized mean ranking (Std.Dev)
Gene-disease	ProphNet	0.9393	0.0609 (0.1597)
LOO	rcNet	0.80572	0.1944 (0.2448)

Gene-disease	ProphNet	0.80717	0.1930 (0.2618)
new associations	rcNet	0.71636	0.2835 (0.2907)

Domain-disease	ProphNet	0.9319	0.0683 (0.1537)
LOO	domainRBF	0.8678	0.1322 (0.2361)

Performance comparison for leave-one-out cross-validation prioritization experiments using OMIM.

### Gene-disease validation with new OMIM associations

Another validation that we have performed is predicting new associations between phenotypes and genes in 387 case studies from new entries added to OMIM between May 2007 and May 2010, since these relationships are not reported in the datasets used in our study. Each case study consists of a phenotype and a set of genes (mostly only one) associated with it. Results of the comparison can be seen in Figure 4. AUC values are shown in Table 1. The results show that our algorithm clearly outperforms rcNet (corrected p-value *<*0.05) predicting new relationships not explicitly represented in the data network.

### Domain-disease validation

To prove that our algorithm not only prioritizes genes, but can prioritize other biological entities, we have performed a leave-one-out domain-disease validation test. For each relation between a domain and a phenotype in our datasets, we run the prioritization methods on a global network in which the direct arc connecting the protein domain and phenotype of interest was removed. The domain of interest was set as the query set and the methods were asked to rank all the phenotypes associated to this query set.

Our method has been compared with domainRBF for this task, since this method has been recently proposed for domain-disease prioritization and builds the phenotype-domain network using the same data sources considered in this study. We set the parameters of domainRBF testing for best performance. A diffusion kernel was selected to compute distances in interactions matrices. *B*_0 _and *V*_0 _were set to 0 and 1, respectively.

**Figure 5:**
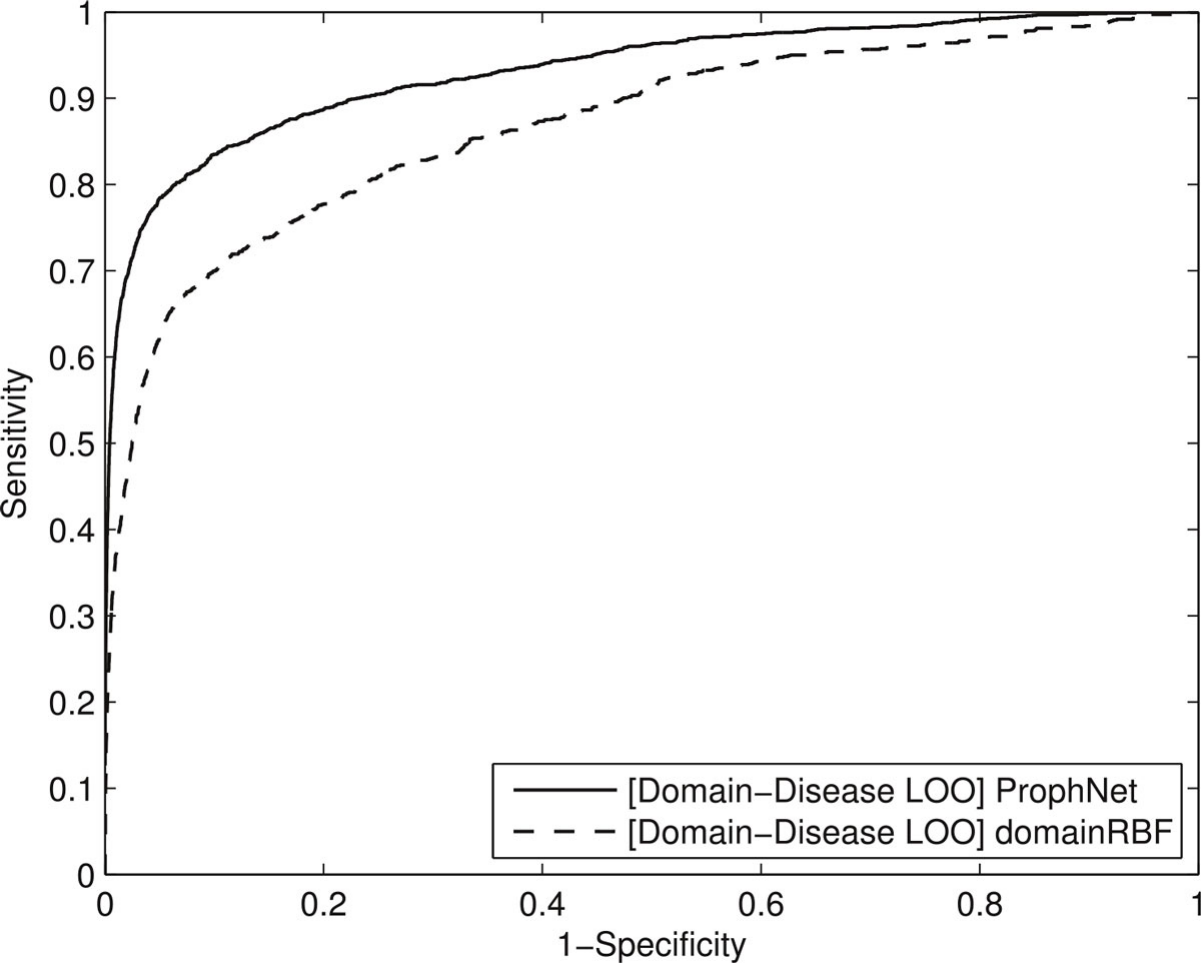
**ROC curves ProphNet vs. domainRBF**. ROC curves for domain-disease prioritizations with ProphNet and domainRBF.

Results show that our method significantly improves the results provided by domainRBF for disease-domain prioritization (corrected p-value *<*0.05). The highest difference in performance is around AUC 10%, which suggests that our method prioritizes more target phenotypes in the top of the ranking. ROC curves for this comparison can be seen in Figure 5.

### Robustness analysis

We carried out several experiments to test the robustness of ProphNet to parameter variation. First, we checked that varying the parameter *α*, which controls the importance of prior information in label propagation, does not significantly affect performance, as previous works suggested for other methods [10,18]. Values ranging between 0.5 and 0.9 reported similar performance for ProphNet, but best result were obtained with *α *set to 0.9.

Second, we tested the impact of varying the parameter *γ *in the results. *γ *was used to limit the propagation of noise in the label propagation between different networks. The experiments showed that, although for any value of *γ *(in [0, 1]) ProphNet reported a good performance, the best results were obtained for *γ *in [0.002, 0.004].

Results from these experiments can be found in Additional file 2.

### Case studies

In order to show the applicability of the proposed method on real case studies, we have used it for gene-disease prioritization of some multifactorial disorders such as Alzheimer Disease, Diabetes Mellitus Type 2 and Breast Cancer, using the data sources described in section *Data sources*. Parameters were set to those which reported better results in the validation experiments (*α *= 0.9, *γ *= 0.00375 and *κ *= 10^*-*5^). A list of the genes ranked in the top positions for each disease are shown in Table 2, together with their assigned score. A detailed discussion about the role of these genes in the associated diseases can also be found in Additional file 4. A full list can be obtained by running the tool at the ProphNet website.

**Table 2:** Ranking positions and assigned scores for top prioritized genes for each case study

Alzheimer Disease (MIM:104300)											
**Gene**	**Rank**	**Score**	**Gene**	**Rank**	**Score**	**Gene**	**Rank**	**Score**	**Gene**	**Rank**	**Score**
APP*	1	0.6639	PSEN1	4	0.1946	CST3	7	0.1511	SNCA	10	0.1276
PSEN2*	2	0.5462	TREM2	5	0.1700	ITM2B	8	0.1468	APOE	11	0.1141
MAPT	3	0.2531	HD/HTT	6	0.1585	TYROBP	9	0.1296	NCSTN	12	0.1114
**Diabetes Mellitus Type 2 (MIM:125853)**											

**Gene**	**Rank**	**Score**	**Gene**	**Rank**	**Score**	**Gene**	**Rank**	**Score**	**Gene**	**Rank**	**Score**
IRS1*	1	0.4744	INSR*	5	0.2950	LEPRE1	9	0.0976	ABCC8	37	0.0456
PPP1R3A*	2	0.4660	TCF1*	6	0.2168	LEPREL4	10	0.0976			
SLC2A4*	3	0.4194	PLN	7	0.1164	NEUROD1	14	0.0905			
IPF1*	4	0.3308	HADHSC	8	0.0976	KCNJ11	30	0.0595			
**Breast Cancer (MIM:114480)**											

**Gene**	**Rank**	**Score**	**Gene**	**Rank**	**Score**	**Gene**	**Rank**	**Score**	**Gene**	**Rank**	**Score**
BRCA1*	1	0.5019	PIK3CA*	5	0.3199	ELAC2	9	0.1038	ATM	13	0.0934
RAD51*	2	0.4919	MSH2	6	0.1636	RAD51AP1	10	0.1031	CHEK2	29	0.0551
BRCA2*	3	0.4813	RB1	7	0.1607	RAD54L	11	0.1031			
NBN/NBS1*	4	0.3547	TP53	8	0.1307	FANCD2	12	0.1017			

Gene symbol, rank position and assigned score for genes in the top of the ranking for each case study. Entries marked with asterisks were directly connected by an arc to the disease of interest in the data network.

#### Results for Alzheimer Disease

Our method was used to prioritize genes related to Alzheimer Disease (MIM:104300). Table 2 shows genes ranked in the top positions which were previously known (OMIM records) to be connected with Alzheimer, such as *APP *and *PSEN2*. Furthermore, new relationships not explicitly reported in OMIM are suggested by analysing other genes in the top 10. For example, *MAPT *was ranked 3th in the obtained prioritized list. This gene provides the instructions for making a protein called *tau *that can be found throughout the nervous system (including neurons of the brain) so it has been associated with Alzheimer [28]. *PSEN1*, with known relations to Alzheimer type 3 [29] was ranked 4th. *TREM2 *was ranked 5th, suggesting an important role in Alzheimer as shown by some population studies [30,31]. *HD*/*HTT *was ranked 6th, and although this gene has not yet been directly associated with Alzheimer, it has been shown to affect Huntington's disease [32]. More details about the other genes in the top 10 are provided in Additional file 4.

#### Results for Diabetes Mellitus Type 2

Our method was used to prioritize genes related to Diabetes Mellitus (DM) Type 2 (MIM:125853). Genes previously known to be connected with the disease, according to OMIM records, are: *IRS1, INSR, IPF1, SLC2A4, PPP1R3A *and *TCF1*, all ranked in the top 6 of the obtained prioritized list of genes. New putative candidate genes were discovered in the top 10. *PLN *(ranked 7th) was not related to Diabetes in the corresponding OMIM entry, however [33] reports a role of PLN in diabetic cardiomyopathy. *HADHSC *was ranked 8th since it has been related to Hyperinsulinemic hypoglycemia [34,35]. The inferred relationship between Diabetes and *LEPRE1 *(ranked 9th) cannot be derived from the published literature and further studies are required to study the possible connections of this gene to DM. Other interesting genes were ranked high, such as *KCNJ11*, ranked 30th, which presents polymorphisms that confer susceptibility to Diabetes mellitus type 2 [36]; or *ABCC8*, ranked 37th, whose mutations increase the risk of diabetes as suggested by [37].

#### Results for Breast Cancer

We performed a prioritization for Breast cancer (MIM:114480). Previously known genes related to this disease according to OMIM are: *BRCA1, RAD51, BRCA2, NBN *and *PIK3CA*, all included in the top 5 returned by ProphNet for this disease.

New relations not explicitly represented in the data network were discovered in the top ranking. Defects in *MSH2 *(ranked 6th) can cause different types of cancer as pointed out by [38]. *RB1 *(ranked 7th) and *TP53 *(ranked 8th) act as tumour suppressors [39]. *ELAC2 *(ranked 9th) has not been associated with breast cancer but with prostate cancer [40]. *RAD51AP1 *(10th) is closely related with *RAD51 *(2nd) [41]. *RAD54L *(11th) plays an important role repairing and recombining DNA in mammalian cells [42]. *FANCD2 *(12th) interacts with the *BRCA1 *and *BRCA2 *genes in the DNA repair process to reduce the risk of breast cancer [43]. *ATM *(13th) has been associated with the disease in various studies [44]. Other relevant genes were found below in the top list, such as *CHEK2 *(ranked 29th), also associated to propensity to suffer breast cancer as shown by [45].

## Conclusion

In this paper we have introduced ProphNet, a novel network-based method that allows to accomplish any prioritization task from a network representing the corresponding data interactions. Our method is flexible and can be run on a graph composed of an arbitrary number of data networks representing biological entities of different type. This paper illustrates how to run ProphNet to perform gene-disease and domain-disease prioritization tasks, and provides experimental evidence that ProphNet outperforms other prioritization algorithms specifically designed for these tasks. A ProphNet web application has also been developed as a result of this work (the user guide can be found in Additional file 5).

The results obtained by ProphNet on real case studies on Alzheimer, DM and Breast Cancer show the potential of the method to suggest putative candidate genes to be involved in a disease. A detailed analysis of the literature also allowed us to validate the results provided by the algorithm, since many of the top ranked genes were already known to be related to the diseases. We consider that prioritization methods are useful for assisting scientists at early research stages and to formulate novel hypotheses of interest.

The extensive experimentation also allowed us to study the indirect influence of considering protein domains for the prioritization of candidate genes and diseases. Results show that the addition of domain interactions produces an obvious improvement with respect to existent algorithms, revealing the importance of this source of information (barely used before in this task). In the future, one of our main goals is to see how our method behaves in other prioritization problems and using different entities and sources of data not covered in this work. Furthermore, we plan to study in more detail the quality of the datasets and their influence on performance, and apply new methods of propagation to try to improve the results.

## List of abbreviations used

*AUC*: Area Under the Curve; *DM*: Diabetes Mellitus; *LOO*: Leave-One-Out; *PPI*: Protein-Protein Interaction; *ROC*: Receiver Operating Characteristic.

## Competing interests

The authors declare that they have no competing interests.

## Authors' contributions

VM developed the ProphNet prioritization algorithm, carried out the experiments and wrote the paper. CC and AB guided and supervised the project and participated in writing the paper. All authors read and approved the final manuscript.

## Supplementary Material

Additional file 1 **Performance comparison using propagation and correlation**. Performance of the obtained results using correlation or propagation in the last step of the algorithm. Click here for file

Additional file 2 **Robustness Analysis Results**. Robustness test results varying *γ *threshold. Click here for file

Additional file 3 **ProphNet execution times and memory usage**. ProphNet execution times and memory usage for the experiments reported in this work. Click here for file

Additional file 4 **Top 50 genes**. ProphNet's top 50 ranked genes for Alzheimer's Disease, Breast Cancer and Diabetes Mellitus Type 2. Click here for file

Additional file 5 **ProphNet User Guide**. ProphNet web tool user guide. Click here for file

## Declarations

Funding for publication of the article was contributed by project TIN2009-13489 of DGICT, Madrid.

This article has been published as part of *BMC Bioinformatics *Volume 15 Supplement 1, 2014: Integrated Bio-Search: Selected Works from the 12th International Workshop on Network Tools and Applications in Biology (NETTAB 2012). The full contents of the supplement are available online at http://www.biomedcentral.com/bmcbioinformatics/supplements/15/S1.
