# Supplementary material for: ProphNet: A generic prioritization method through propagation of information
Source: BMC Bioinformatics. 2014 Jan 10;15(Suppl 1):S5. doi: 10.1186/1471-2105-15-S1-S5 (PMC4015146; doi:10.1186/1471-2105-15-S1-S5)
Supplement: Additional file 1 — Performance comparison using propagation and correlation. Performance of the obtained results using correlation or propagation in the last step of the algorithm. [file 1471-2105-15-S1-S5-S1.pdf]

PROPAGATION AND CORRELATION ANALYSIS RESULTS

(GENE-DISEASE LEAVE-ONE-OUT VALIDATION)

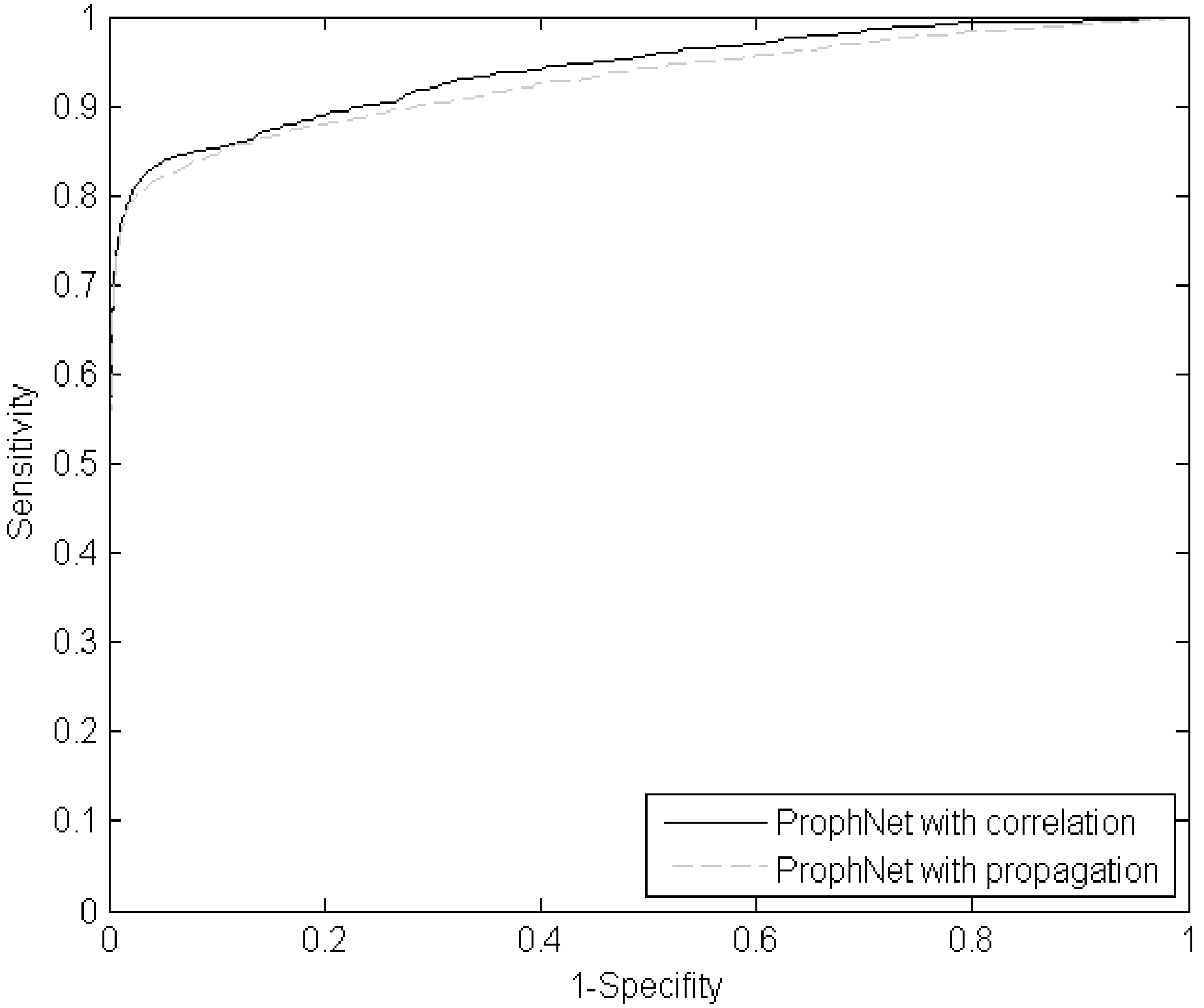

Method	Correlation	Propagation
**AUC**	0.9393	0.9277
**Mean ranking**	309	368
**Standard deviation**	811	944
