# Supplementary material for: ProphNet: A generic prioritization method through propagation of information
Source: BMC Bioinformatics. 2014 Jan 10;15(Suppl 1):S5. doi: 10.1186/1471-2105-15-S1-S5 (PMC4015146; doi:10.1186/1471-2105-15-S1-S5)
Supplement: Additional file 2 — Robustness Analysis Results. Robustness test results varying γ threshold. [file 1471-2105-15-S1-S5-S2.pdf]

ROBUSTNESS ANALYSIS RESULTS
(GENE-DISEASE VALIDATION WITH NEW OMIM ASSOCIATIONS)

Gamma	AUC	Normalized mean ranking (Std. Dev)
0.0005	0.79834	0.2018 (0.2613)
0.0010	0.80599	0.1942 (0.2604)
0.0020	0.80792	0.1922 (0.2615)
0.0030	0.80749	0.1927 (0.2621)
0.0040	0.80685	0.1933 (0.2619)
0.0050	0.80669	0.1935 (0.2617)
0.0100	0.80526	0.1949 (0.2619)
0.0300	0.79982	0.2003 (0.2644)
0.0500	0.79684	0.2033 (0.2649)
0.1000	0.7916	0.2086 (0.2666)
0.5000	0.78169	0.2185 (0.2696)
1.0000	0.78169	0.2185 (0.2696)
