# Supplementary figures and images for: ProphNet: A generic prioritization method through propagation of information
Source: BMC Bioinformatics. 2014 Jan 10;15(Suppl 1):S5. doi: 10.1186/1471-2105-15-S1-S5 (PMC4015146; doi:10.1186/1471-2105-15-S1-S5)

# PROPHNET EXECUTION TIMES AND MEMORY USAGE

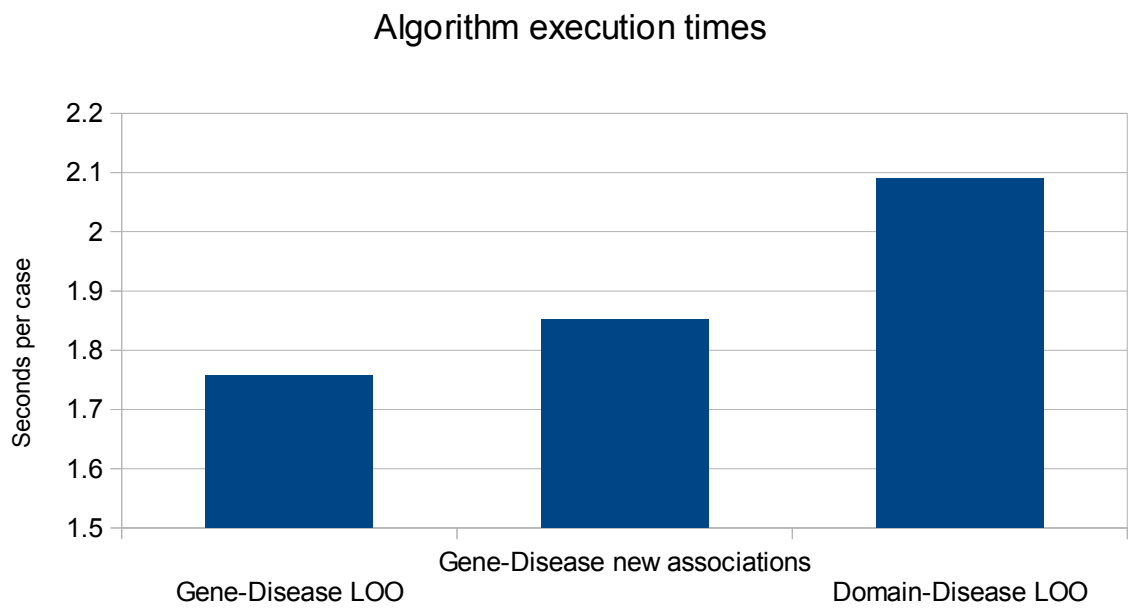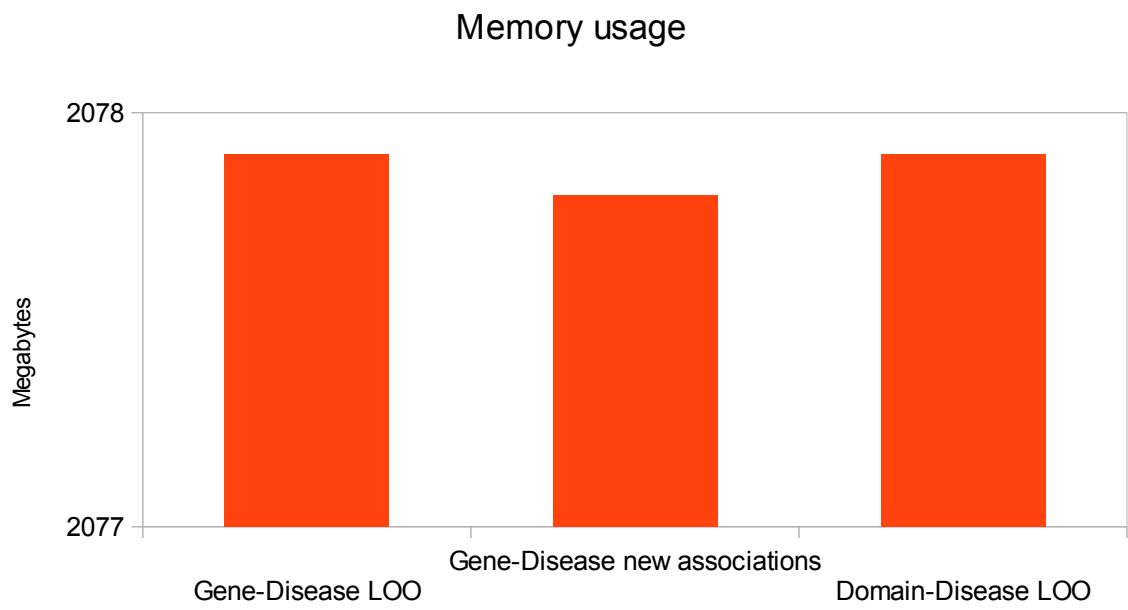

Supplement: Additional file 3 — ProphNet execution times and memory usage. ProphNet execution times and memory usage for the experiments reported in this work. [file 1471-2105-15-S1-S5-S3.pdf]
