# Supplementary material for: ProphNet: A generic prioritization method through propagation of information
Source: BMC Bioinformatics. 2014 Jan 10;15(Suppl 1):S5. doi: 10.1186/1471-2105-15-S1-S5 (PMC4015146; doi:10.1186/1471-2105-15-S1-S5)
Supplement: Additional file 4 — Top 50 genes. ProphNet's top 50 ranked genes for Alzheimer's Disease, Breast Cancer and Diabetes Mellitus Type 2. [file 1471-2105-15-S1-S5-S4.pdf]

**TOP 50 ALZHEIMER (MIM:104300) GENES PRIORITIZED BY PROPHNET**

| Rank | Gene | Score | Rank | Gene | Score |
|---|---|---|---|---|---|
| 1 | APP | 0.66395 | 26 | SIRPB1 | 0.069677 |
| 2 | PSEN2 | 0.54624 | 27 | KCNIP4 | 0.067622 |
| 3 | MAPT | 0.25306 | 28 | CHMP2B | 0.066392 |
| 4 | PSEN1 | 0.19459 | 29 | ICAM5 | 0.06531 |
| 5 | TREM2 | 0.17 | 30 | APBB3 | 0.063825 |
| 6 | HD | 0.15854 | 31 | APBA3 | 0.061983 |
| 7 | CST3 | 0.15113 | 32 | PRNP | 0.06149 |
| 8 | ITM2B | 0.14676 | 33 | COL25A1 | 0.059462 |
| 9 | TYROBP | 0.1296 | 34 | HADHB | 0.05945 |
| 10 | SNCA | 0.12759 | 35 | CASP6 | 0.056407 |
| 11 | APOE | 0.11406 | 36 | KCNIP3 | 0.055112 |
| 12 | NCSTN | 0.11144 | 37 | SNCB | 0.054846 |
| 13 | PSENEN | 0.09454 | 38 | KIR2DS2 | 0.054442 |
| 14 | APH1A | 0.09454 | 39 | NOTCH3 | 0.052885 |
| 15 | APH1B | 0.093457 | 40 | KLRC3 | 0.052401 |
| 16 | METTL2B | 0.091739 | 41 | PRSS3 | 0.05117 |
| 17 | HADH2 | 0.088582 | 42 | CHRNA7 | 0.050067 |
| 18 | SPON1 | 0.086666 | 43 | APBB2 | 0.04729 |
| 19 | TM2D1 | 0.086666 | 44 | NOTCH4 | 0.046163 |
| 20 | BACE2 | 0.086666 | 45 | CTNND2 | 0.044223 |
| 21 | DOCK3 | 0.077497 | 46 | SERPINI1 | 0.043768 |
| 22 | CD300E | 0.069677 | 47 | FLNB | 0.043587 |
| 23 | CLEC5A | 0.069677 | 48 | CASP8 | 0.043244 |
| 24 | NCR2 | 0.069677 | 49 | APPBP1 | 0.042937 |
| 25 | TREM1 | 0.069677 | 50 | CTSD | 0.042268 |

**TOP 50 DIABETES MELLITUS TYPE II (MIM: 125853) GENES PRIORITIZED BY PROPHNET**

| Rank | Gene | Score | Rank | Gene | Score |
|---|---|---|---|---|---|
| 1 | IRS1 | 0.4744 | 26 | PHIP | 0.062078 |
| 2 | PPP1R3A | 0.46597 | 27 | ATP2A3 | 0.062078 |
| 3 | SLC2A4 | 0.41937 | 28 | ATP2A1 | 0.062029 |
| 4 | IPF1 | 0.33078 | 29 | ACOX1 | 0.060055 |
| 5 | INSR | 0.29497 | 30 | KCNJ11 | 0.059551 |
| 6 | TCF1 | 0.21682 | 31 | C1QTNF5 | 0.059257 |
| 7 | PLN | 0.11641 | 32 | - | 0.050414 |
| 8 | HADHSC | 0.097584 | 33 | ATP2A2 | 0.050161 |
| 9 | LEPRE1 | 0.097584 | 34 | STRN3 | 0.048852 |
| 10 | - | 0.097584 | 35 | ARF3 | 0.048383 |
| 11 | MLSTD2 | 0.097584 | 36 | SLN | 0.048282 |
| 12 | FAM62B | 0.097584 | 37 | ABCC8 | 0.04558 |
| 13 | IDH2 | 0.097584 | 38 | PSMD7 | 0.044634 |
| 14 | NEUROD1 | 0.090461 | 39 | MVP | 0.043341 |
| 15 | PCSK1 | 0.077833 | 40 | SNF1LK2 | 0.042414 |
| 16 | SLC2A2 | 0.075549 | 41 | EHD2 | 0.042374 |
| 17 | TCF2 | 0.074199 | 42 | PPARG | 0.041591 |
| 18 | MAFA | 0.070823 | 43 | PCSK1N | 0.041119 |
| 19 | PDIA6 | 0.070718 | 44 | HK2 | 0.04051 |
| 20 | FKBP10 | 0.069568 | 45 | BPY2IP1 | 0.0403 |
| 21 | KBTBD10 | 0.067907 | 46 | SPOP | 0.039407 |
| 22 | DLD | 0.066068 | 47 | ENPP1 | 0.038238 |
| 23 | ARFIP1 | 0.064772 | 48 | MFRP | 0.038163 |
| 24 | RPS6KA1 | 0.064346 | 49 | RAB7 | 0.037582 |
| 25 | IAPP | 0.062876 | 50 | GATA5 | 0.036751 |

### TOP 50 BREAST CANCER (MIM:114480) GENES PRIORITIZED BY PROPHNET

| Rank | Gene | Score | Rank | Gene | Score |
|---|---|---|---|---|---|
| 1 | BRCA1 | 0.50185 | 26 | TREX1 | 0.060285 |
| 2 | RAD51 | 0.49193 | 27 | HMG20B | 0.058471 |
| 3 | BRCA2 | 0.4813 | 28 | MRE11A | 0.057521 |
| 4 | NBN | 0.3547 | 29 | CHEK2 | 0.055145 |
| 5 | PIK3CA | 0.31986 | 30 | CDK4 | 0.054203 |
| 6 | MSH2 | 0.16361 | 31 | ERCC2 | 0.052391 |
| 7 | RB1 | 0.16072 | 32 | BAP1 | 0.051401 |
| 8 | TP53 | 0.13068 | 33 | MSH6 | 0.051175 |
| 9 | ELAC2 | 0.10385 | 34 | - | 0.050248 |
| 10 | RAD51AP1 | 0.10305 | 35 | MLH1 | 0.049023 |
| 11 | RAD54L | 0.10305 | 36 | MUTYH | 0.048841 |
| 12 | FANCD2 | 0.10167 | 37 | RPA1 | 0.048761 |
| 13 | ATM | 0.093381 | 38 | C17orf28 | 0.048712 |
| 14 | RNASEL | 0.083185 | 39 | TP53BP1 | 0.048516 |
| 15 | BCCIP | 0.0778 | 40 | AXIN2 | 0.048076 |
| 16 | SHFM1 | 0.077161 | 41 | SMC1L1 | 0.047339 |
| 17 | DCLRE1C | 0.076188 | 42 | BUB1B | 0.046962 |
| 18 | C11orf30 | 0.075987 | 43 | FANCG | 0.04636 |
| 19 | BLM | 0.074494 | 44 | RAD52 | 0.04634 |
| 20 | DMC1 | 0.070191 | 45 | ATRX | 0.045625 |
| 21 | MDC1 | 0.069038 | 46 | STK11 | 0.044099 |
| 22 | RAD50 | 0.065818 | 47 | BCL2 | 0.042166 |
| 23 | H2AFX | 0.06564 | 48 | EXO1 | 0.041624 |
| 24 | ATR | 0.065553 | 49 | GABRB1 | 0.04145 |
| 25 | PTEN | 0.060815 | 50 | RET | 0.040784 |
