# Supplementary material for: ProphNet: A generic prioritization method through propagation of information
Source: BMC Bioinformatics. 2014 Jan 10;15(Suppl 1):S5. doi: 10.1186/1471-2105-15-S1-S5 (PMC4015146; doi:10.1186/1471-2105-15-S1-S5)
Supplement: Additional file 5 — ProphNet User Guide. ProphNet web tool user guide. [file 1471-2105-15-S1-S5-S5.pdf]

PROPHNET WEB TOOL USER GUIDE

ProphNet web tool allows users to perform prioritizations of genes-diseases-proteins domains. The tool has been designed to be easy to use. ProphNet does not require any registration or identification for use. When we enter prophnet we see the following screen.

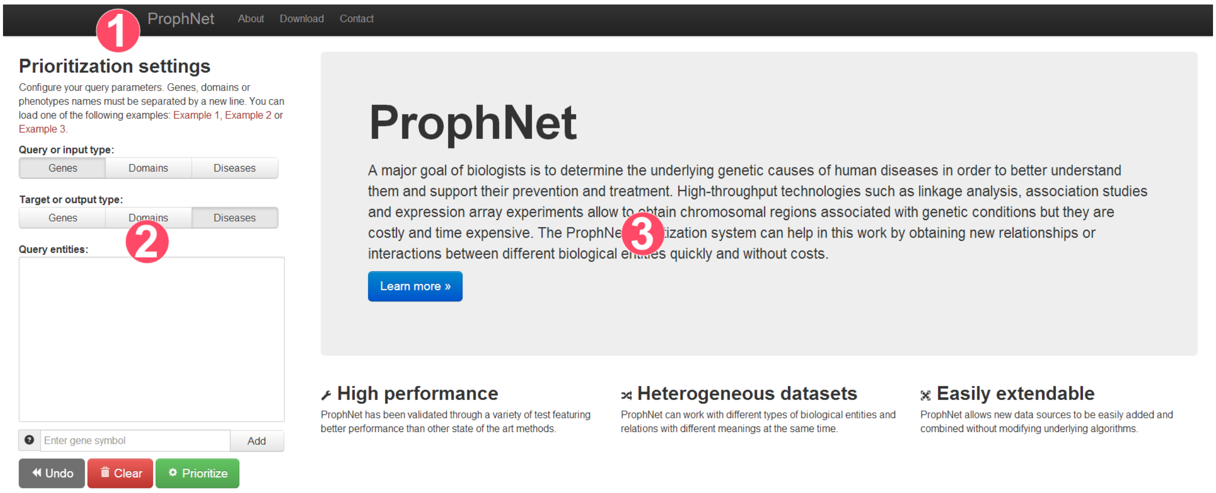

We can see three different parts in the interface. The action bar **[1]** allows direct access to a number of interesting additional information about ProphNet and Matlab source code. The prioritization settings **[2]** area is the space where the user sets the parameters of prioritization to be performed. Finally, the work space **[3]**, which initially only contains information about ProphNet, is the area where the user will see the results of the prioritization.

Users interact with the tool using prioritization settings area. This area can be seen in more detail in the following figure.

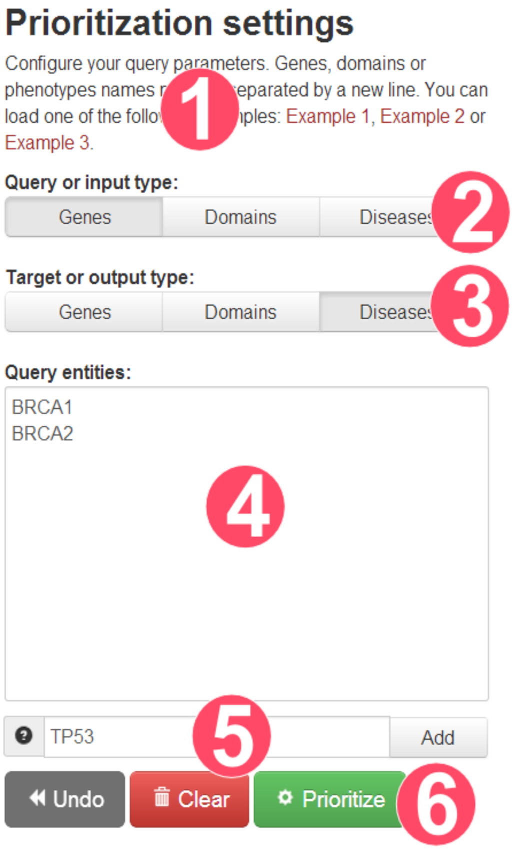

This area contains multiple elements. The first one, is a help text **[1]**. This text explains what type of input is expected. The user can also load some examples clicking on the links. To configure a prioritization the user first have to specify a query or input type **[2]** and a target or output type **[3]**. With the configuration of the figure, the user would introduce genes identifiers and would obtain a list of ranked diseases. The input list **[4]** is the component where the user introduces the list of identifiers of the elements for the query. Each element must be introduced in a new line. To help users to input correct names, an auto-complete component is available **[5]**. Based on user input, this component will suggest matching names. For details on what types of identifiers are supported, the user can press the button with the exclamation mark. Finally, some action buttons **[6]** are provided. After entering prioritization settings the user must press the *Prioritize* button. While performing prioritization the following dialog will be displayed.

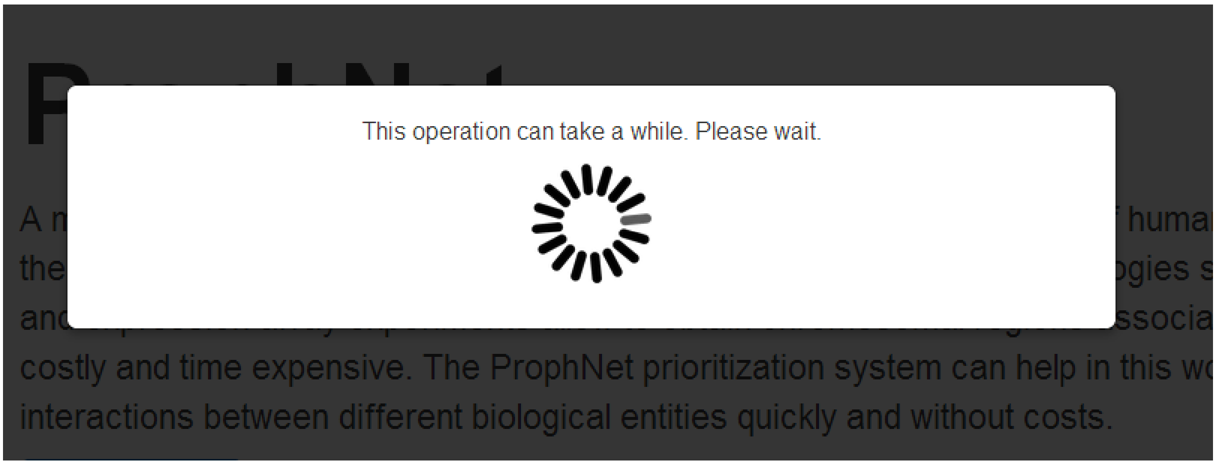

Prioritization can take several minutes depending on the server load. After this wait, results are shown as the following figure.

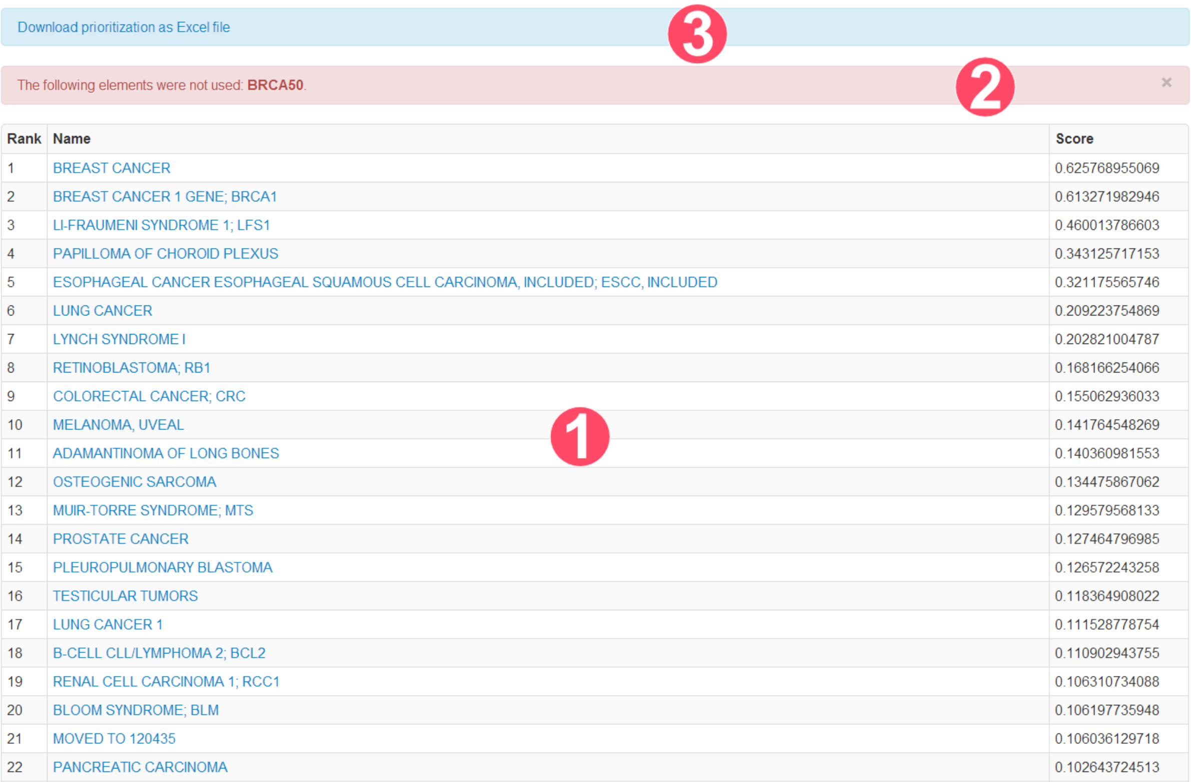

Rank	Name	Score
1	BREAST CANCER	0.625768955069
2	BREAST CANCER 1 GENE; BRCA1	0.613271982946
3	LI-FRAUMENI SYNDROME 1; LFS1	0.460013786603
4	PAPILLOMA OF CHOROID PLEXUS	0.343125717153
5	ESOPHAGEAL CANCER ESOPHAGEAL SQUAMOUS CELL CARCINOMA, INCLUDED; ESCC, INCLUDED	0.321175565746
6	LUNG CANCER	0.209223754869
7	LYNCH SYNDROME I	0.202821004787
8	RETINOBLASTOMA; RB1	0.168166254066
9	COLORECTAL CANCER; CRC	0.155062936033
10	MELANOMA, UVEAL	0.141764548269
11	ADAMANTINOMA OF LONG BONES	0.140360981553
12	OSTEOGENIC SARCOMA	0.134475867062
13	MUIR-TORRE SYNDROME; MTS	0.129579568133
14	PROSTATE CANCER	0.127464796985
15	PLEUROPULMONARY BLASTOMA	0.126572243258
16	TESTICULAR TUMORS	0.118364908022
17	LUNG CANCER 1	0.111528778754
18	B-CELL CLL/LYMPHOMA 2; BCL2	0.110902943755
19	RENAL CELL CARCINOMA 1; RCC1	0.106310734088
20	BLOOM SYNDROME; BLM	0.106197735948
21	MOVED TO 120435	0.106036129718
22	PANCREATIC CARCINOMA	0.102643724513

We can see different parts in the results area. The ranked table **[1]** is the essential part. This table shows the rank, the name or identifier and the assigned score to each element. Names can be clicked for more details about each entry. The elements in the red bar **[2]** are those that have been excluded from the query due to not being in ProphNet database. Finally, blue bar **[3]** lets users download results as Excel files.
